# Globalization of precision medicine programs in lung cancer: a health system challenge

**DOI:** 10.1016/j.lanepe.2023.100819

**Published:** 2023-12-12

**Authors:** Christian Rolfo, Valeria Denninghoff

**Affiliations:** a Center for Thoracic Oncology, The Tisch Cancer Institute, Icahn School of Medicine at Mount Sinai, New York City, NY, USA; b Liquid Biopsy and Cancer Interception Group, Centre for Genomics and Oncological Research - Pfizer - University of Granada - Andalusian Regional Government (GENyO), Granada, Spain; c Molecular-Clinical Lab - University of Buenos Aires (UBA) - National Council for Scientific and Technical Research (CONICET), Argentina

Precision medicine is an innovative approach to disease prevention and treatment that considers differences in people's genes, injuries, environments, and lifestyles to target the right therapies to the right patients at the right time. In oncology, precision medicine uses genetic and molecular information, tailoring treatment on the single patient profile, optimizing efficacy and minimizing toxicities.^1^ This approach is revolutionizing lung cancer diagnosis and treatment. However, despite widely adopted, its benefit in clinical practice still remains to be fully elucidated.

To clarify this aspect, objectively assessing the benefits to patients is necessary. In this issue of the *Lancet Regional Health—Europe*, Kästner et al.^2^ investigated the effectiveness of the German Network Genomic Medicine (GNGM) in terms of overall survival (OS) using nationwide real-world data. The GNGM is a precision medicine program that provides comprehensive and high-quality multiplex molecular diagnostics and standardized, personalized treatment recommendations for patients with advanced non-small cell lung cancer.

They compared the treatment and overall survival (OS) of patients with (n = 509) and without (n = 7213) GNGM participation, with a minimum follow-up period of six months. Patients participating in the GNGM had a significantly improved OS compared to the non-GNGM group (median OS: 10.5 months vs. 8.7 months, *p* = 0.008, HR = 0.84, 95% CI: 0.74–0.95). The use of approved tyrosine kinase inhibitors (TKI) in the first-line setting was significantly higher in the GNGM group than in the non-GNGM group (GNGM: 8.4% vs. non-GNGM: 5.1%, *p* = 0.001). Overall, patients receiving first-line TKI treatment had significantly higher one-year OS rates than patients treated with PD-1/PD-L1 inhibitors and/or chemotherapy (67.2% vs. 40.2%, *p* < 0.001). In conclusion, patients receiving broad NGS-based molecular diagnostics and treatment decision support had significantly higher OS rates than patients receiving routine clinical care based on the clinician's choice.^2^

It would be interesting to globalize this concept to evaluate the cost-benefit of the new techniques and target drugs so that the increase in cost that precision medicine entails does not collapse healthcare systems, not only for developed countries but also for low and middle-income countries (LMIC). Unfortunately, more than half of the world's population still has no access to precision medicine and cannot reap the benefits.^3^ Furthermore, access to NGS varies widely across countries and a recent analysis across European countries revealed that less than 10% of the specimens requiring molecular testing are analysed with NGS-based techniques,^4^ potentially limiting the access to precision medicines in a large proportion of eligible patients.

To include personalized medicine within health systems, it is necessary to evaluate differential research needs for decision-making—i.e., economic evaluations, preparation, delivery, sustainability, and scale-up. The World Economic Forum's Precision Medicine Programme aims to identify and scale up solutions for more resilient, efficient, and equitable healthcare systems to deliver the best possible care and keep people across societies and nations healthy. It supports the building and testing policy frameworks to realize the benefits of precision medicine for society while reducing risks.^5^

Technology is transforming the world of healthcare and paving the way for a better future. Precision medicine is a term widely used to denote the application of scientific processes, technology, and evidence to improve the care of patients by optimizing the therapeutic benefit of interventions to treat, manage, cure and ideally prevent human diseases. Often, this involves matching a sophisticated understanding of disease mechanisms and pathophysiology with highly specific diagnostic, therapeutic and/or preventive measures to improve clinical outcomes in selected patients or populations. There are conceptional and structural challenges with implementing personalized medicine, including educating physicians on the benefits and limitations of genomic profiling, cooperation between physicians and pathologists, timely reporting of testing results, clinical implications of the comprehensive genomic profile, the composition of multidisciplinary tumour boards, access to adequate therapies, clinic-genomic databases, and bioinformatics.^6^ Multidisciplinary molecular tumour board are essential for adequate interpretation and management of the large body of genomic information provided by NGS,^7^ fostering clinical trials enrolment and access to targeted therapies. Multicentre networks, such as GNGM, might increase testing rates and patient clinical trials access, reducing disparities between institutions and regions.

Looking into the future, all these progresses will be overtaken by artificial intelligence that is outpacing itself daily, integrated with an aging informatics infrastructure that will be fed with higher resolution data from technologies combined with digital imaging and personal devices. Precision Medicine will have to expand following practical principles, local customs, different governmental structures, regulatory environments, and efficient use of resources. With new policy approaches to understand the gaps, a future can be envisioned and help take substantial steps towards its implementation on a global scale.

## Contributors

Dr Rolfo & Denninghoff: conceptualisation, supervision, writing—original draft, and writing—review & editing.

## Declaration of interests

Dr. Rolfo has received speaker honoraria from AstraZeneca, Roche and MSD, advisory board honoraria from Inivata, Archer, Boston Pharmaceuticals, MD Serono and Novartis, Bayer, Invitae, Regeneron, Bostongene, Scientific Advisory Board member of Imagene, and institutional research funding from LCRF-Pfizer and NCRF, non-renumerated research support from Guardant Health and Foundation Medicine. He has non-renumerated leadership roles at the International Society of Liquid Biopsy (ISLB), the International Association for Study of Lung Cancer (IASLC), the European School of Oncology (ESO), and Oncology Latin American Association (OLA). Dr. Denninghoff reports non-renumerated leadership roles at the International Society of Liquid Biopsy (ISLB) and the International Association for Study of Lung Cancer (IASLC).
